# Blood Catalase, Superoxide Dismutase, and Glutathione Peroxidase Activities in Alcohol- and Opioid-Addicted Patients

**DOI:** 10.3390/medicina61020204

**Published:** 2025-01-24

**Authors:** Nino Asatiani, Nelly Sapojnikova, Tamar Kartvelishvili, Lali Asanishvili, Nestan Sichinava, Zaza Chikovani

**Affiliations:** 1 Elevter Andronikashvili Institute of Physics, Ivane Javakhishvili Tbilisi State University, Tbilisi 0179, Georgia; nelly.sapojnikova@tsu.ge (N.S.); tamar.kartvelishvili@tsu.ge (T.K.);; 2 Narcological Clinic “Nishati”, Tbilisi 0186, Georgiazazachik@gmail.com (Z.C.)

**Keywords:** alcohol, opioids, catalase, superoxide dismutase, glutathione peroxidase, intoxication, withdrawal

## Abstract

*Background and Objectives*: Multiple evaluations of oxidative stress in individuals with substance use disorder show elevated levels compared to non-substance-abusing individuals. Information concerning antioxidant defense mechanisms in relation to alcohol and opioid dependence is variable and sometimes contradictory. The objective of the present investigation was to identify and compare several antioxidants in plasma during distinct phases of alcohol and opioid dependency (intoxication and withdrawal). *Materials and Methods*: This case study focuses on individuals with opioid and alcohol addiction. We recruited 80 participants (males aged 40 ± 10 years) and equally divided them into two categories: those with alcohol addiction and those with opioid addiction. A control group consisted of 20 healthy adults (males aged 35 ± 10 years). The spectrophotometric methods were used to quantify catalase, superoxide dismutase (SOD), and glutathione peroxidase (GPx) activity in plasma. Antioxidant values were analyzed between groups using pairwise Mann–Whitney tests. *Results*: During withdrawal from alcohol and opioids, catalase activity tends to decrease compared to intoxication. The overall activity of superoxide dismutase exhibited an increase during alcohol intoxication and withdrawal and a reduction during opioid withdrawal compared to the intoxication phase. Both alcohol and opioids reduced plasma GPx activity in withdrawal cases, although the extent of this decrease varied considerably. *Conclusions*: The study confirms the valuable impact of addiction on the organism’s oxidative stress and reveals various behaviors of antioxidant defense enzymes during intoxication and withdrawal phases.

## 1. Introduction

Recent studies have highlighted the critical involvement of oxidative stress in multiple neurodegenerative conditions, especially those more common in the aging population, such as Alzheimer’s and Parkinson’s diseases, as well as stroke [1,2]. Neurodegenerative diseases of unknown origin, characterized by a complex interplay of genetic, environmental, and lifestyle factors, consistently exhibit oxidative stress [3]. It remains unclear whether oxidative stress induces degenerative processes, or rather, results from them. High oxygen levels make the central nervous system especially susceptible to oxidative damage. Neurons contain significant quantities of redox-active transition metals, which can catalytically generate reactive oxygen species (ROS) [4]. Neurons are particularly vulnerable to oxidative damage due to their relatively low antioxidant activity when compared to other cells and tissues [3,5].

All cellular macromolecules, including lipids, carbohydrates, proteins, and polynucleotides, are susceptible to damage from an excess of reactive oxygen species. Such damage may lead to the formation of secondary products that can be significantly more harmful than the initial ROS [6]. Cells may generate the following reactive oxygen species: hydroxyl radicals, superoxide anion radicals, hydrogen peroxide, and singlet oxygen, among others. The hydroxyl radical is the most detrimental, believed to be the principal contributor to oxidative stress damage. All bio-macromolecules react with it at rates controlled by diffusion or at nanoscale distances from its formation, triggering a chain reaction of harmful byproducts and damages [7].

The body’s biological defense against free radicals can be categorized into two levels. At the first level, there are components that serve to inhibit the endogenous generation of free radicals. Vitamins C and E, together with glutathione (GSH), are considered the principal endogenous non-enzymatic antioxidants [8]. They can neutralize reactive oxygen species before the onset of lipid peroxidation. Moreover, vitamin C has the potential to regenerate vitamin E. In protecting membrane fatty acids from lipid peroxidation, vitamin E is the most effective antioxidant. Its effect of decreasing lipid peroxidation has neuroprotective benefits [9].

The capture and elimination of already formed free radicals take place at the second, or main, level of antioxidant defense [8,10]. An effective antioxidant cascade, including both enzyme-based and non-enzyme-based mechanisms, controls damages caused by ROS. Superoxide dismutase, catalase, and the glutathione system, which consists of glutathione reductase and glutathione peroxidase, are among the primary enzymatic antioxidants [11]. Superoxide dismutase catalytically breaks down the superoxide anion radical into oxygen and hydrogen peroxide (H_2_O_2_). The enzymes catalase and glutathione peroxidase (GPx), which uses glutathione (GSH) as an electron donor, are the most efficient in removing hydrogen peroxide. Glutathione reductase is the enzyme that converts oxidized glutathione (GSSG) back into GSH. Glutathione peroxidases play a crucial role in the reduction of hydroperoxides (ROOH).

Lipid peroxidation, protein oxidation, and DNA oxidation are the three primary ways that reactive oxygen species induce age-related cellular damage. Severe cerebral ischemia has been associated with the degradation of membrane phospholipids [4,12]. Patients with neurodegenerative disorders exhibit elevated levels of oxidative stress markers in their brain tissues [13]. The areas of the brain affected in stroke victims’ autopsies demonstrated immunocytochemical evidence of oxidative damage to bio-macromolecules [14].

Imbalances in reactive oxygen species and antioxidant defense have been identified as a potential contributor to the toxicity affecting both the central and peripheral nervous systems in individuals with substance use disorder (SUD) [15].

Chronic ethyl alcohol (ethanol) consumption can lead to an addiction known as alcohol dependency. The oxidative metabolism of ethanol takes place in the body due to the involvement of a unique enzyme system in its oxidation [16]. It has been proven that even low doses of alcohol alter the function of regulatory membrane proteins involved in nerve impulse transmission by inducing oxidative stress [17]. Alcohol’s pathogenic effects are attributed to various factors such as the liquefaction and increased “membrane fluidity”, neuronal death, particularly in the cerebral cortex, hypothalamus, and cerebellum, an excess of neurotransmitters affecting the postsynaptic membrane [18], and exaggerated functional stress on dopaminergic nuclei neurons [19]. Chronic ethanol consumption increases the levels of reactive oxygen species in cells, significantly impacting the efficacy of the body’s antioxidant defense system [20,21].

The metabolism of opioids in the body also generates reactive oxygen species. Individuals with opioid use disorder exhibit enduring dysfunctions in brain activity, persisting even after the cessation of opioid consumption. Free radical reactions that cause lipid peroxidation may play a significant role in the development of physical problems linked to opioid addiction [15,22]. Numerous studies have looked at the enzymatic antioxidant capacity of people with neurodegenerative disorders and stroke. These studies have focused on the activities of superoxide dismutase (SOD), catalase, and glutathione peroxidase (GPx) in the blood as the main antioxidants that control oxidative stress [23].

The simultaneous detection of several antioxidants in blood at different stages of alcohol and opioid addiction (intoxication and withdrawal) may improve the early diagnosis of neurodegenerative disorders. This study examined the activity of plasma antioxidants (catalase, SOD, and GPx) in oxidative stress resulting from alcohol and opioid addiction.

## 2. Materials and Methods

### 2.1. Clinical Study

The subjects of this case study were individuals with opioid or alcohol addictions who attended treatment at the private narcological clinic “Nishati”, located in Tbilisi, Georgia. The clinic provides specialized and confidential services, focusing solely on the outpatient management of addiction. Patients do not present with other acute or chronic medical conditions. Each patient underwent the necessary outpatient evaluations at a multidisciplinary clinic, which included a complete blood count, liver function assessments, abdominal ultrasound examinations, coagulation tests, and consultations with a cardiologist.

Eighty participants with alcohol and opioid addictions were recruited, all meeting the appropriate eligibility criteria. The cohort consisted of males with an average age of approximately 40 ± 10 years, and the research specifically focused on this male population. The participants were categorized into two main groups: those with an alcohol addiction and those with an opioid addiction. Each group was further divided into two subgroups: (1) intoxicated state and (2) withdrawal state, with an equal number of matched patients in each subgroup. Additionally, a control group of 20 healthy adults (males aged 35 ± 10 years) was included, all of whom had no history of drug or alcohol abuse and had abstained from alcohol consumption for the preceding month.

Inclusion criteria specified that participants must exclusively use opioid drugs (buprenorphine, methadone, or heroin) without engaging in the use of other psychoactive substances. The age range across the different study cohorts was consistent, and the private clinic served patients from middle and upper socioeconomic classes.

Exclusion criteria related to oxidative stress as contributing factors to the disease context included: a history of acute stroke, active malignancy within the past five years, diabetes, heart failure, liver failure, and any major surgery within the last six months.

The International Classification of Diseases (ICD) established criteria for diagnosing alcohol and drug addiction in the participants. Physicians relied on anamnestic data and clinical measurements, including mydriasis, rhinorrhea, diarrhea, increased lacrimation, hyperhidrosis, arthralgia, myalgia, hypertension, and tachycardia, to determine the state of withdrawal. The quantities of alcohol and narcotics were determined using urine as a biological material. Patients experiencing opioid addiction in both intoxicated and withdrawal states had urine containing methadone, buprenorphine, and heroin. The clinic provides voluntary, anonymous placements (treatments).

Upon admission to the clinic, blood samples from patients with alcohol and opioid addiction were collected using heparinized sterile tubes (lithium heparin, Italy). Blood samples were centrifuged for 10 min at 3000 rpm in order to carry out further plasma analysis. The plasma had been stored at a temperature of −20 °C. The activity of the analyzed antioxidant enzymes in each new sample batch was assessed within one week. Two months at −20 °C is the maximum plasma storage time that does not impact the enzyme activities based on the results obtained.

### 2.2. Human Ethics

According to Georgia law, the act of signing the participation form represents informed consent. The chairman of the National Council on Bioethics at Tbilisi State Medical University authorized the consent forms. The Ivane Javakhishvili Tbilisi State University Research and Development Service, along with the National Council on Bioethics at Tbilisi State Medical University, which supervises the ethical evaluation of biomedical research, validated all experimental protocols (protocol number #8-2022/101, on 22 December 2022).

### 2.3. Quantification of Catalase Activity in Plasma by the Spectrophotometric Method

The spectrometric assessment of catalase activity relies on the enzyme’s capacity to oxidize hydrogen peroxide, as provided by the method of Beers and Sizer [24]. The catalase activity was assessed by quantifying the rate of H_2_O_2_ (7.5 mM) breakdown in a 50 mM potassium phosphate buffer (pH 7.0) with plasma present.

We have established the necessary parameters to measure plasma catalase activity. The protocol is as follows: 0.025 mL of plasma was combined with 2.275 mL of potassium phosphate buffer (50 mM, pH 7.0) at 25 °C. Then, 0.655 mL of hydrogen peroxide was added to achieve a final concentration of 7.5 mM and initiate the process. We carried out the reaction at 25 °C using a NanoDrop2000c spectrophotometer (Fisher Scientific, Waltham, MA, USA) cuvette. For 180 s, the optical density at 240 nm was recorded every 30 s; ε_H2O2_ = 43.6 M^−1^ cm^−1^. We serially diluted the plasma at 1:10, 1:25, and 1:50 using potassium phosphate buffer (50 mM, pH 7.0) prior to measurement. The unit of measurement for catalase activity was U/mL.

### 2.4. Quantification of SOD Activity in Plasma by the Spectrophotometric Method

Total SOD activity in plasma has been measured using a Superoxide Dismutase (SOD) assay kit (Catalog No. abx096009; Abbexa, Cambridge, UK) based on colorimetric superoxide radical detection.

NBT-diformazan is generated from NBT and superoxide by xanthine oxidase (XOD). SOD reduces superoxide concentration, hence reducing the rate of NBT-diformazan formation. The activity of superoxide dismutase is determined by measuring the absorbance at 450 nm, which quantifies NBT-diformazan concentrations. The absorbance was measured using the SmartReader96 (Accuris™ Instruments, Edison, NJ, USA). In this study, plasma was diluted in a ratio of 1:3 with 0.9% NaCl. The SOD assay quantifies the activity of total superoxide dismutase (Cu,Zn-SOD, Mn-SOD, and extracellular SOD) in plasma. The SOD activity was calculated in U/mL.

### 2.5. Quantification of GPx Activity in Plasma by the Spectrophotometric Method

A glutathione peroxidase (GPx) assay kit (Catalog No. abx294002; Abbexa, UK) utilizing colorimetric detection has been employed to measure GPx activity in plasma. The spectrometric assessment of GPx activity relies on the capacity of glutathione peroxidase to convert GSH (reduced monomeric glutathione) into GSSG (glutathione disulfide). The absorbance was measured at 415 nm following the instructions provided by the manufacturer using the SmartReader96 (Accuris™ Instruments, USA). For this study, plasma was diluted 1:2 with deionized distilled water. The GPx activity was calculated and represented in units (U).

### 2.6. Methods of Analysis

In Origin for Windows, version OriginPro9, the antioxidant values were presented as means, medians, and standard deviations. Antioxidant values were analyzed between groups using pairwise Mann–Whitney tests: https://www.socscistatistics.com/tests/mannwhitney/default2.aspx (accessed on 8 January 2025). *p* values below 0.05 were regarded as statistically significant; values below 0.01 indicated a substantial difference; and values above 0.05 were not considered statistically significant. The confidence interval (CI) was calculated at a confidence level of 95% (https://www.omnicalculator.com/statistics/confidence-interval) (accessed on 8 January 2025).

## 3. Results

### 3.1. Plasma Catalase Activity

**Figure 1:**
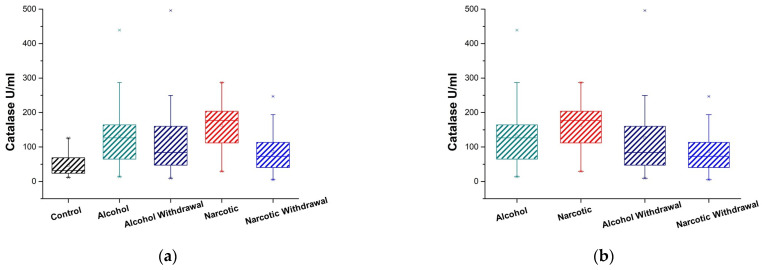
Catalase activity in plasma of substance-dependent patients. (**a**) Comparison of alcohol intoxication (Alcohol) and withdrawal (Alcohol Withdrawal) phases, opioid intoxication (Narcotic) and withdrawal (Narcotic Withdrawal) phases, and control group (Control); (**b**) comparison of stages of alcohol and opioid intoxication and withdrawal with one another.

Plasma levels of catalase activity were measured in patients with alcohol and opioid addictions in both the intoxicated and withdrawal states. Figure 1a and Table 1 and Table 2 present the results for alcohol and opioid addiction and controls.

During alcohol intoxication, catalase activity increases compared to the control, and it remains elevated throughout the period of withdrawal; however, it has a tendency to decrease in comparison to intoxication.

The effect of enzyme activation is particularly noticeable when opioid intoxication occurs. During intoxication, catalase activity nearly triples compared to the control and approaches the control following withdrawal, indicating a dramatic decline but not a return to the control level.

In cases of drug dependence, the levels of catalase activity exhibit considerable and consistent differences between intoxication and withdrawal states (Table 3).

Figure 1b and Table 2 and Table 3 provide data for a comparison of catalase activity between alcohol and opioid addictions, both in the intoxicated state and during withdrawal. The comparison of intoxicated states in alcohol and drug dependency shows that both dependencies demonstrate high catalase activity; however, catalase levels in the drug-intoxicated state are consistently higher with statistically reliable differences between them. In instances of withdrawal, both examined dependents exhibit a notable reduction in catalase activity, with no statistically reliable differences between them.

### 3.2. Plasma SOD Activity

**Figure 2:**
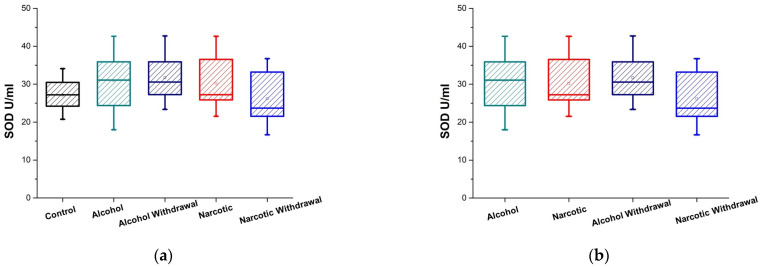
SOD activity in plasma of substance-dependent patients. (**a**) Comparison of alcohol-intoxicated (Alcohol) and withdrawal (Alcohol Withdrawal) phases, opioid-intoxicated (Narcotic) and withdrawal (Narcotic Withdrawal) phases, and control group (Control); (**b**) comparison of stages of alcohol and opioid intoxication and withdrawal with one another.

Patients suffering from alcohol and opioid addictions had their plasma levels of total superoxide dismutase activity assessed when they were intoxicated and during withdrawal, and controls. Figure 2a displays and Table 1 and Table 2 summarize the results related to alcohol and opioid addiction and controls. The activity of total SOD, encompassing Cu, Zn-SOD, Mn-SOD, and extracellular SOD, shows an increase compared to the control only in the context of alcohol, during both intoxication and withdrawal. In alcohol dependency, both intoxication and withdrawal stages have elevated SOD activity levels that are comparable to one another; however, opioid withdrawal tends to decrease.

Total SOD activity is not changed in comparison to the controls in the opioid intoxication state (Figure 2a). In instances of drug withdrawal, SOD activity exhibits a considerable decrease relative to the drug intoxication state.

Upon examining the total SOD activity in the plasma of individuals addicted to alcohol or opioids, as depicted in Figure 2b and Table 2 and Table 3, both addictions exhibit elevated levels of activity, like catalase, with minimal differences between them during intoxication. In instances of withdrawal, the examined SOD activity in patients with drug dependency is markedly lower than this with alcohol dependency.

### 3.3. Plasma GPx Activity

**Figure 3:**
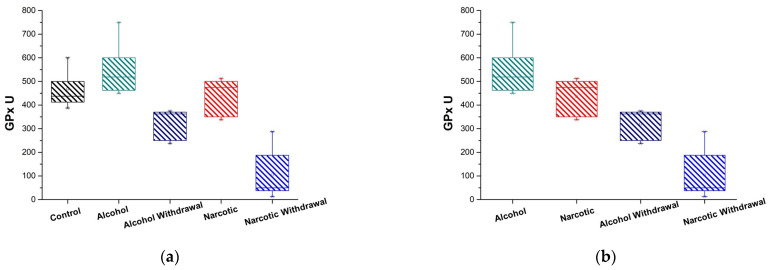
GPx activity in plasma of substance dependent patients. (**a**) Comparison of alcohol-intoxicated (Alcohol) and withdrawal (Alcohol Withdrawal) phases, opioid-intoxicated (Narcotic) and withdrawal (Narcotic Withdrawal) phases, and control group (Control); (**b**) comparison of stages of alcohol and opioid intoxication and withdrawal with one another.

Figure 3a illustrates the GPx activity in the plasma of alcohol- and opioid-dependent individuals during intoxication and withdrawal phases and controls. The results represented in Figure 3a and Table 1 and Table 2 indicate that during intoxication, the activity of glutathione peroxidases remains comparable to the control; however, during withdrawal, there is a substantial decrease in their activity relative to both the control and the intoxicated state (Table 3).

An analogous pattern of peroxidase activity is noted in individuals with drug addiction and healthy control (Figure 3a). Withdrawal is marked by a serious reduction in GPx activity relative to the control and intoxication (Table 2).

In terms of the comparison of peroxidase activity in the plasma of patients who are dependent on alcohol and opioids, as demonstrated in Figure 3b, the alcohol intoxication state is characterized by a higher level of GPx activity than the opioid intoxication state. In withdrawal cases, both alcohol and opioids significantly diminish plasma GPx activity; however, the reduction differs enormously. Specifically, the decrease in drug withdrawal is nearly five times greater than the decrease in alcohol withdrawal.

## 4. Discussion

Under normal physiological conditions, all regulatory mechanisms are set to guarantee that an elevation in reactive oxygen species stimulates an enhancement in antioxidant system activity, thereby restoring free radical levels to baseline. The disruption of regulatory mechanisms in pathological conditions leads to an uncontrolled elevation of reactive oxygen species, culminating in oxidative stress (OS). Free radicals interact with macromolecules, causing damages that result in a reduction or total loss of functional activity. The regulation of ROS levels is executed by specialized systems that defend against the deleterious effects of free radicals and preserve the overall balance. Homeostasis in the body is maintained by antioxidants, including both non-enzymatic and, in cases of significant oxidative stress, enzymatic forms. Superoxide dismutase, glutathione peroxidase, glutathione reductase, and catalase constitute the fundamental endogenous enzymatic defense mechanisms. They promote protection by immediately eliminating radicals and transforming them into less reactive species [10]. The superoxide anions and hydroxyl radicals are neutralized by superoxide dismutase and glutathione; singlet oxygen is mitigated by tocopherols and ubiquinone; the peroxide radicals are minimized by carotenoids; hydrogen peroxide is decomposed by catalase and glutathione peroxidase; and hydroperoxides can be eliminated by the glutathione peroxidase/glutathione reductase system. These enzymes eliminate radical intermediates and may prevent other oxidation reactions by undergoing self-oxidation [10].

Neurons are especially vulnerable to the detrimental effects of ROS because of the high content of fatty acids and their peroxidation, high metabolic rates, reduced antioxidant levels, and lower regeneration potential. At the same time, OS produces conformational and structural alterations in proteins, intensifying oxidative damage. In neurons, oxidized proteins accumulate in the cytoplasm, resulting in the formation of Aβ plaques that sustain oxidative injury through increased levels of reactive oxygen species [25,26]. Oxidative stress can affect brain cells and their metabolism across all levels (nucleic acids, proteins, lipids, and carbohydrates), resulting in their damage [1,27].

Alcohol dependency is a pathology characterized by the regular consumption of ethanol, which not only elevates reactive oxygen species levels in cells but also substantially disrupts the functional efficacy of the body’s antioxidant system [28,29]. Numerous research studies have established that the activities of superoxide dismutase, catalase, and glutathione peroxidases in plasma/serum, neurons, and erythrocytes are distinct in individuals experiencing alcohol abuse throughout intoxication and withdrawal phases compared to non-abused individuals [30,31,32]. Literature data confirm the substantial influence of alcohol consumption on the activity of blood antioxidant enzymes; however, an unambiguous conclusion regarding its direction cannot be drawn, as this may vary based on factors such as the patient’s age, duration of chronic alcohol intake, quantity and quality of alcohol consumed, dietary quality, somatic disorder, medication, and others [33,34].

The pathological process of developing opioid dependency, using natural and synthetic opioid alkaloids, indicates that their metabolism may contribute to the generation of free radicals [15]. Chronic consumption of drugs results in significant oxidative damage and neuroinflammation in the brain [35]. Furthermore, the low levels of the antioxidant enzyme catalase and glutathione contribute to the increased susceptibility of neurons to the damaging effects of oxidative stress [36].

The results obtained in this study reveal oxidative stress and a disruption in the plasma enzymatic antioxidant system, occurring during both intoxication and withdrawal in instances of alcohol and opioid dependence. Moreover, during the transition from intoxication to withdrawal in opioid dependence, there is an increase in oxidative potential within the cell, primarily involving catalase, which should notably influence the stability of essential intracellular components and, consequently, the redox processes during this transition. The activity of plasma total superoxide dismutase (SOD), encompassing Cu, Zn-SOD, Mn-SOD, and extracellular SOD, constitutes a component of the primary defense mechanism. Its activity is markedly elevated relative to the control only in cases of alcohol, including both intoxication and withdrawal. In cases of narcotic dependence, the total SOD activity in all cases does not significantly differ from the control group. GPx is a peculiar antioxidant because, in addition to sharing the substrate H_2_O_2_ with catalase, it is also capable of reacting effectively with lipids and other organic hydroperoxides. Furthermore, it is an active participant in the glutathione cycle, which is a critical component of antioxidant defense. GPx functions as an antioxidant by using GSH as a cofactor. GSH is a recognized neuroprotective agent. Several chronic diseases, including neurodegenerative disorders, have recognized glutathione depletion in their progression. A sharp reduction in GPx during withdrawal in drug-dependent individuals signals a substantial decline in plasma GSH, thereby putting at risk the entire glutathione cycle, which is a crucial neuroprotective pathway. On the other hand, the significant decrease in glutathione peroxidase activity during withdrawal in drug-dependent individuals allows us to propose a specific form of oxidation during the withdrawal state, associated with conformational changes in proteins linked to signaling pathways in oxidative damage. The accumulation of unfolded or misfolded proteins in neuronal cells can lead to neurodegenerative disorders. The results obtained suggest that the oxidative stress associated with alcohol and opioid dependency may possess different natures and, hence, have different contributions to the predisposition to neurodegenerative diseases.

## 5. Conclusions

Alcohol and opioid dependency, encompassing both intoxication and withdrawal, are identified by alterations in the activity of enzymatic antioxidants. The shift from intoxication to withdrawal in alcohol and opioid dependence is marked by a significant change in the activity of antioxidant enzymes, especially concerning the glutathione-dependent antioxidant enzyme (GPx). In the case of opioid withdrawal, GPx has dropped about five times more than in the case of alcohol withdrawal. Along with medical indicators, this can serve as a biochemical indicator of the state of withdrawal. We speculate that a specific oxidative state during opioid withdrawal links to signaling pathways involved in oxidative damage and subsequent neurodegenerative disorders.

## 6. Limitations

The research is focused on the male demographic due to specific national characteristics. In Georgia, the prevalence of alcohol and opioid dependence among women is statistically lower than that among men, and their frequency of medical consultations is significantly less. The study has several limitations, including its focus on a single geographic region (Tbilisi, Georgia), a limited sample size, and the absence of multivariate statistical analyses.

## Figures and Tables

**Table 1:** Comparison of plasma catalase, SOD, and GPx activities’ *p*-values between patients in alcohol-intoxicated (Alcohol) and withdrawal (Alcohol Withdrawal) states, opioid-intoxicated (Narcotic) and withdrawal (Narcotic Withdrawal) states, and healthy volunteers (Control).

Antioxidant Enzyme Activity	Control and Alcohol	Control and Alcohol Withdrawal	Control and Narcotic	Control and Narcotic Withdrawal
Catalase U/mL	0.0001 (are significantly different *p* < 0.01)	0.0058 (are significantly different *p* < 0.01)	0.00001 (are significantly different *p* < 0.01)	0.034 (are marginally significant *p* < 0.05)
SOD U/mL	0.038 (are marginally significant *p* < 0.05)	0.028 (are marginally significant *p* < 0.05)	0.20 (are not significantly different *p* ≥ 0.05)	0.55 (are not significantly different *p* ≥ 0.05)
GPx U	0.20 (are not significantly different *p* ≥ 0.05)	0.012 (are significantly different at *p* < 0.05)	0.76 (are not significantly different *p* ≥ 0.05)	0.008 (are significantly different *p* < 0.01)

**Table 2:** Confidence interval (CI) for the plasma antioxidant enzymes in the case of healthy volunteers and substance-dependent patients. Values are presented as means ± margin of error; (numbers in the parentheses are CI).

Enzymes	Control	Alcohol	Alcohol Withdrawal	Narcotic	Narcotic Withdrawal
Catalase U/mL	46.13 ± 18.05 = (28.07, 64.18)	131.87 ± 22.60 = (109.27, 154.47)	126.41 ± 42.49 = (83.92, 168.89)	162.30 ± 28.79 = (133.51, 191.09)	87.30 ± 24.28 = (63.02, 111.58)
SOD U/mL	27.121 ± 2.13 = (24.99, 29.25)	30.91 ± 2.311 = (28.59, 33.22)	31.71 ± 2.49 = (29.22, 34.19)	30.18 ± 2.55 = (27.62, 32.73)	26.18 ± 3.19 = (22.98, 29.37)
GPx U	465.7 ± 74.55 = (392.95, 542.05)	550 ± 91.89 = (458.11, 641.89)	319 ± 60.46 = (258.54, 379.46)	435 ± 74.07 = (360.93, 509.07)	104.17 ± 87.35 = (16.82, 191.51)

**Table 3:** Comparison of plasma catalase, SOD, and GPx activities’ *p*-values between addicted patients in alcohol-intoxicated (Alcohol) and withdrawal (Alcohol Withdrawal) states, and opioid intoxicated (Narcotic) and withdrawal (Narcotic Withdrawal) states.

Antioxidant Enzyme Activity	Alcohol and Alcohol Withdrawal	Narcotic and Narcotic Withdrawal	Alcohol and Narcotic	Alcohol Withdrawal and Narcotic Withdrawal
Catalase U/mL	0.33 (are not significantly different *p* ≥ 0.05)	0.00094 (are significantly different *p* < 0.01)	0.044 (are marginally significant *p* < 0.05)	0.29 (are not significantly different *p* ≥ 0.05)
SOD U/mL	0.67 (are not significantly different *p* ≥ 0.05)	0.023 (are marginally significant *p* < 0.05)	0.93 (are not significantly different *p* ≥ 0.05)	0.0093 (are significantly different *p* < 0.01)
GPx U	0.008 (are significantly different *p* < 0.01)	0.008 (are significantly different *p* < 0.01)	0.27 (are not significantly different *p* ≥ 0.05)	0.023 (are marginally significant *p* < 0.05)

## Data Availability

The data are available from the corresponding author.
